# Nasal Carriage of Methicillin‐Resistant *Staphylococcus aureus* Among Paramedical Students at Pokhara University, Nepal

**DOI:** 10.1155/ipid/5571214

**Published:** 2025-12-26

**Authors:** Alina Sharma, Mandira Chhusyabaga, Bhawana Baral, Binita Dhakal, Sabina Bhurtyal, Suresh Jaiswal

**Affiliations:** ^1^ Department of Microbiology, School of Health and Allied Sciences, Pokhara University, Pokhara, Nepal, pu.edu.np

**Keywords:** D-test, MRSA, nasal carriage, paramedical students

## Abstract

**Background:**

Methicillin‐resistant *Staphylococcus aureus* (MRSA) has been the most common cause of community‐acquired infections and nosocomial infections worldwide. A higher risk of nosocomial infections is associated with colonization of the anterior nares of paramedical trainees who are continuously exposed to MRSA. The purpose of this study is to ascertain the prevalence of MRSA nasal carriage in paramedical students, as well as the antibiogram and inducible clindamycin resistance patterns.

**Method:**

Nasal swabs were taken from 246 paramedical students enrolled in Pokhara University’s School of Health and Allied Sciences in Nepal. Following the isolation of *S. aureus* from nasal swabs, bacteria were tested for antibiotic susceptibility using a modified Kirby–Bauer disc diffusion test. A cefoxitin disc (30 μg) was used to identify MRSA, and the *D*‐test was carried out in accordance with CLSI standards. SPSS version 25 was utilized for data collection and analysis.

**Result:**

MRSA accounted for 80 (36.7%) of the 217 *Staphylococcus aureus* isolates, while 137 (63.3%) were methicillin‐sensitive *S. aureus* (MSSA). The total nasal carriage rate of MRSA was reported to be 32.5% (80/246), and males (46.0%) and those over 30 years old (55.6%) had rates of MRSA that were higher, respectively. There were 112 (51.6%) multidrug‐resistant (MDR) isolates, but all isolates were vancomycin‐sensitive. Inducible clindamycin resistance was found in 17.1% of isolates according to the D‐test.

**Conclusion:**

MRSA and *S. aureus* nasal carriage rates were high among paramedical students; therefore, care must be taken to prevent nosocomial infections brought on by MRSA nasal carriage.

## 1. Introduction

Gram‐positive, cluster‐forming, spherical bacteria, *Staphylococcus* are members of the Micrococcaceae family [1]. It was initially noted in 1871 in pyogenic lesions in humans [2]. In addition to being present in the environment*, Staphylococcus aureus* is a normal human flora that is found in skin and mucous membranes, primarily on the nasal surface of the healthy people [3]. Due to the prevalence of infections in both community and hospital settings, treatment is still difficult since multidrug‐resistant (MDR) strains such as methicillin‐resistant *S. aureus* (MRSA) are becoming more prevalent [4]. Pneumonia, endocarditis, and bacteremia are among the diseases caused by this opportunistic infection [5].

Bloodstream infections, infections of the bones, joints, skin, and soft tissues, and other clinical problems are all caused by *S. aureus* [6]. Treatment of infections has become more difficult since the advent of MDR forms such as MRSA (2). It is challenging to deal with MRSA since these microbes have become resistant to the available medications [7]. Macrolides, lincosamides, and streptogramin B (MLSB) are a few of the therapy choices for infections linked to MRSA. Along with being helpful for individuals who are allergic to penicillin, clindamycin is a good substitute, particularly for infections of the skin and soft tissues [8]. The most often found resistance mechanism in gram‐positive organisms is MLSB resistance, which can be either constitutive or inducible [7].

The mecA gene found on the bacterial chromosome of MRSA is part of a larger genetic element known as the staphylococcal chromosomal cassette mec (SCCmec) region, which is responsible for providing resistance to various antibiotics depending on the specific SCCmec type present [9]. The mecA gene produces a specific protein known as PBP2a, a penicillin‐binding protein (PBP) with low affinity for penicillin. Resistance to MLSB is caused by the methylation of the binding site of 23S rRNA, which is controlled by a gene called erm. Inducible MLSB resistance, also known as iMLSB, can be identified through the D‐zone test [8]. Clindamycin therapy may fail clinically if iMLSB resistance cannot be detected [10]. Because inducible clindamycin resistance is so common, it is critical to accurately identify it to avoid therapeutic failure in infections brought on by these strains [11].

MDR is defined as nonsusceptibility to at least one agent in three or more antimicrobial categories. *Staphylococcus aureus* is resistant to beta‐lactam antibiotics, such as methicillin, which are frequently used to treat *Staphylococcus aureus* infections. In addition to resistance to methicillin, the strain may also be resistant to other classes of antibiotics such as macrolides (e.g., erythromycin), tetracyclines (e.g., doxycycline), clindamycin, fluoroquinolones (e.g., ciprofloxacin), aminoglycosides (e.g., gentamicin), and vancomycin (in some cases, especially with vancomycin‐resistant *S. aureus*, or VRSA) [12]. By gaining resistance genes, particularly the mecA gene, which codes for a modified PBP2a that inhibits cell wall formation and prevents beta‐lactams from binding, MRSA has evolved to evade the effects of these medications. [13].

The combination of horizontal gene transfer, antibiotic‐induced selection, and bacterial evolution results in highly resistant and hazardous infections, as demonstrated by the link between MRSA and MDR (14). When determining MDR status for *Staphylococcus aureus*, the following classes: β‐lactams (penicillin, oxacillin, and cefoxitin), macrolides (erythromycin and azithromycin), lincosamides (clindamycin), aminoglycosides (gentamicin and amikacin), fluoroquinolones (ciprofloxacin and levofloxacin), tetracyclines (tetracycline and doxycycline), sulfonamides (trimethoprim and sulfamethoxazole), glycopeptides (vancomycin and teicoplanin), are used [13]. Among the *Staphylococcus aureus* isolates, a substantial correlation between methicillin resistance and MDR was found. The majority of MRSA isolates showed resistance to several different classes of antibiotics, suggesting that MRSA strains have a far higher MDR incidence than methicillin‐sensitive *S. aureus* (MSSA) strains [14]. The results highlight the fact that coresistance to non–β‐lactam antibiotics such as macrolides, fluoroquinolones, aminoglycosides, and sulfonamides is often present alongside methicillin resistance, suggesting that MRSA isolates are more likely to be MDR [15].

The treatment choices for MRSA infections are significantly reduced by this co‐occurrence, which also emphasizes the critical necessity for routine surveillance [16]. Identifying MRSA carriers quickly and accurately is critical since previous research raises the likelihood that medical and paramedical students/HCWs contribute significantly to MRSA transmission [10]. Global variation can be seen in *S. aureus* nasal carriage among the overall adult population [11]. Due to variables such as hygiene, antibiotic use, and healthcare practices, the nasal carriage of *S. aureus,* a major reservoir for MRSA, differs throughout the world [17]. A study conducted at Dessie Referral Hospital found that 28.8% of healthcare workers carried *S. aureus*, with 12.7% of all healthcare workers being MRSA carriers [18]. A study found that 45% of clinical students in China were carriers of *S. aureus* [19]. Healthcare professionals in Brazil and Argentina have a 42.9% *S. aureus* carriage rate and a 12.3% MRSA carriage rate [20].

On the basis of colonization persistence and bacterial load, human nasal carriers of *S. aureus are* classified as persistent carriers, intermittent (transient) carriers, and noncarriers. Persistent carriers have a high bacterial load (> 10^3^ CFU per swab); it is mostly seen in hospital/immunocompromised patients. Intermittent (transient) carriers have comparatively lower bacterial load than persistent carriers, while noncarriers are rarely or never colonized [21].

Numerous studies have revealed that the knowledge of the frequency of nasal carriage of *S. aureus* and MRSA, along with their current antimicrobial profile, becomes necessary in the selection of appropriate treatment options for these carriers.

## 2. Methods

This laboratory‐based cross‐sectional study was performed in the Department of Microbiology, School of Health and Allied Sciences, Pokhara University, Nepal, among 246 paramedical students during a period of 4 months (January 2023–April 2023).

### 2.1. Inclusion and Exclusion Criteria

Included participants were paramedical students from Pokhara University’s School of Health and Allied Sciences who consented to participate in the study after being directly exposed to clinical samples during their clinical postings at Pokhara Academy of Health Sciences. Due to their frequent exposure to clinical settings and handling of clinical samples, which raises the possibility of coming in touch with possible *S. aureus* and MRSA carriers, these students were particularly selected. To prevent confounding variables that might affect nasal carriage rates, students with upper respiratory tract infections or those who had recently taken antibiotics were not included in the study.

### 2.2. Sample Collection

Nasal swabs were collected using a commercially available sterile cotton swab. The swab was inserted into the nasal cavity approximately 1–2 cm and then rotated clockwise and anticlockwise for 3‐4 turns before being withdrawn. This process was repeated for both nostrils using the same swab for each specimen.

### 2.3. Experimental Protocol

#### 2.3.1. Identification of *S. aureus*

Nasal swabs were inoculated into mannitol salt agar and incubated at 37°C for 24 h. Isolates were identified by examination of colony characteristics, gram staining, catalase test, slide coagulase test, tube coagulase test, and deoxyribonuclease test.

#### 2.3.2. Antibiotic Susceptibility of *Staphylococcus aureus* and Identification of MRSA, VRSA, and MDR

An antibiotic susceptibility test using a modified Kirby–Bauer disc diffusion method was performed on isolates from the samples. The HiMedia antibiotic discs were kept in a refrigerator between 2°C and 8°C and were firmly sealed to keep out light and moisture. Prior to susceptibility testing, the ATCC reference strain of *S. aureus* 25,923 was used to maintain quality control (QC) of the antibiotics. In addition, the lot number, expiration date, and appropriate storage were ensured. The zone of inhibition according to CLSI guidelines was examined and confirmed. The following antibiotics were utilized in the study: erythromycin (15 μg), clindamycin (2 μg), amikacin (30 μg), cefoxitin (30 μg), vancomycin (30 μg), ciprofloxacin (5 μg), tetracycline (30 μg), and amoxicillin (10 μg). According to CLSI criteria, the result was regarded as “sensitive” and “resistant.”

Using a cefoxitin (30 μg) disc as suggested by CLSI guidelines, MRSA was identified. The inhibitory zone diameter of MSSA was found to be ≥ 22 mm, while that of MRSA was reported to be ≤ 21 mm.

#### 2.3.3. Detection of iMLSB

The D‐test was used to check for inducible resistance in the isolates. According to CLSI’s recommendation, a 0.5‐McFarland equivalent suspension of *S. aureus* was plated into MHA. On the MHA, CD (2 μg) and *E* (15 μg) discs were positioned 15 mm from the center. The plates were then incubated for 18 h at 37°C before being examined. Inducible resistance was not present in the organism if the *D* zone measured less than 13 mm and the CD zone measured more than 21 mm, both of which were round in shape (D‐test negative). A positive D‐test result indicated that the organism had inducible resistance if the *D* zone was less than 13 mm and the CD zone was greater than 21 mm, with a D‐shaped zone surrounding the CD.

### 2.4. Data Analysis

All the obtained data were entered in MS Excel, and the analysis was carried out by SPSS Version 25 (IBM Corporation, Armonk, NY, USA). The chi‐square test was applied to determine the association between different variables, and the *p* value of < 0.05 was considered statistically significant.

## 3. Results

Out of 246 nasal swabs collected from paramedical students, *Staphylococcus aureus* was isolated from 217 (88%) students. Among them, 80 (36.9%) were found to be MRSA carriers. Overall, nasal carriage of MRSA was found to be (80/246) 32.5%.

### 3.1. Demographic Characteristics of the Study Population

#### 3.1.1. Distribution of *S. aureus* by Gender

**Figure 1:**
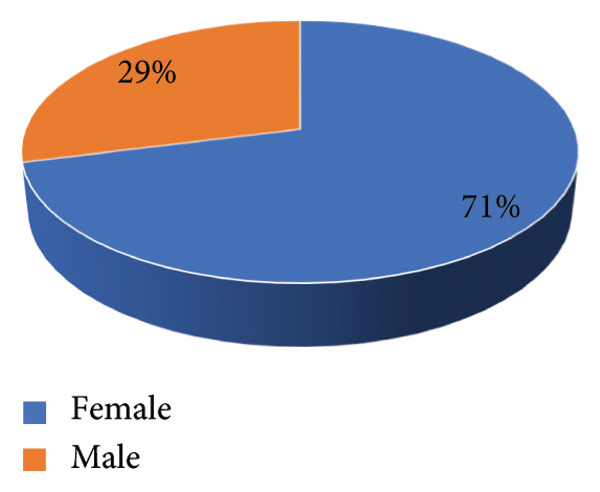
Distribution of *S. aureus* by gender.

The nasal carriage rate of *Staphylococcus aureus* was higher in females compared to that of males (71% vs. 29%), as shown in Figure 1.

#### 3.1.2. Distribution of *S. aureus* by Age

**Figure 2:**
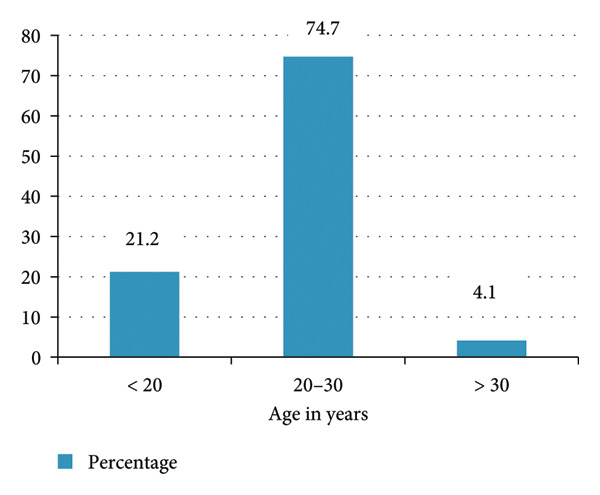
Distribution of *S. aureus* by age.

*S. aureus* carriage rate was found to be 46 (21.2%) in the age group less than 20 years, 162 (74.7%) between 20 and 30, and 9 (4.1%) in those more than 30 years. A higher carriage rate of *S. aureus* was observed in students aged 20–30 than in other age groups, which is shown in Figure 2.

#### 3.1.3. Distribution of *S. aureus* by Formation and Degree

The nasal carriage rate among nursing students was higher (48), followed by the BSc. MLT (43), BPH (42), B. Pharm [22], MPH [19], MSc. MM and MB [15], BPT [23], and M. Pharm [7], as shown in Table 1.

**Table 1:** Distribution of *S. aureus* by formation and degree.

Formation and degree	Frequency	Percentage (%)
BNS	48	22.1
B. Pharm	25	11.5
BPH	42	19.4
BPT	14	6.5
BSc. MLT	43	19.8
M. Pharm	7	3.2
MPH	21	9.7
MSc MM and MB	17	7.8
Total	217	100

### 3.2. Antibiotic Susceptibility Pattern of *S. aureus*

**Figure 3:**
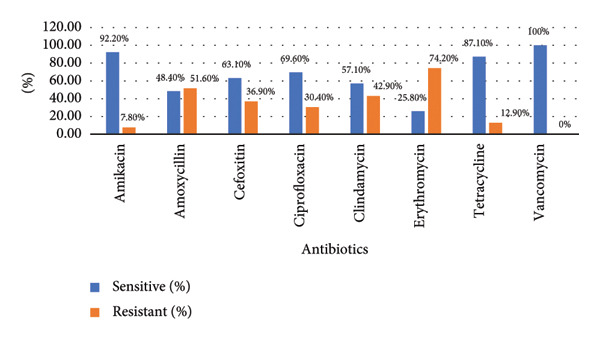
Antibiotic susceptibility pattern of *S. aureus.*

As illustrated in Figure 3, the susceptibility profile of the bacterial isolates against eight commonly used antibiotics reveals marked variability in both sensitivity and resistance rates. Notably, all isolates (100%) were sensitive to vancomycin. High sensitivity was also observed for amikacin (92.2%) and tetracycline (87.1%). In contrast, susceptibility to amoxicillin was only 48.4%, and resistance rates were correspondingly high. Intermediate levels of sensitivity were seen for cefoxitin (63.1%), ciprofloxacin (69.6%), and clindamycin (57.1%). The most concerning finding is for erythromycin, which exhibited the highest resistance (74.2%), with only 25.8% of isolates being sensitive.

#### 3.2.1. Distribution of MRSA

**Figure 4:**
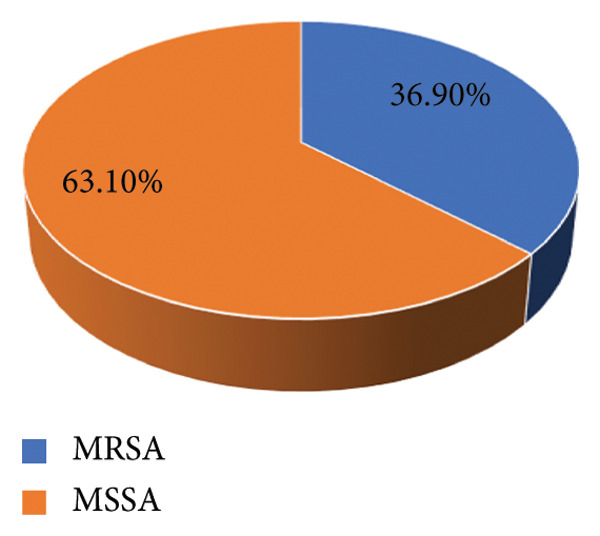
Distribution of MRSA.

Distribution of *Staphylococcus aureus* isolates showing that 36.9% were MRSA and 63.1% were MSSA as shown in Figure 4.

#### 3.2.2. Distribution of MDR

**Figure 5:**
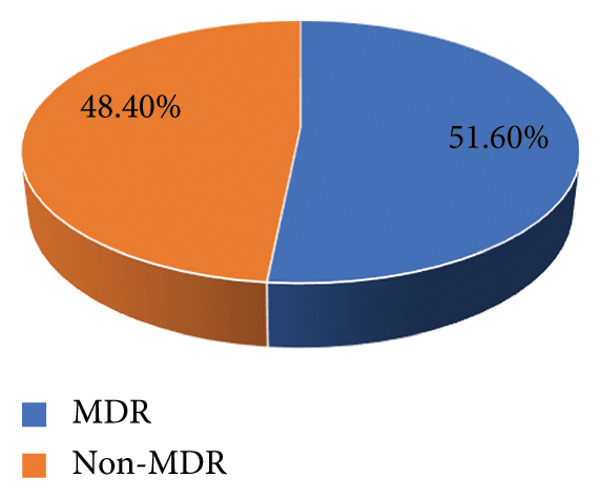
Distribution of MDR.

Among the isolates, 51.6% (112/217) and 48.4% (105/217) were found to be MDR and non‐MDR, respectively, which is represented in Figure 5.

#### 3.2.3. Distribution of iMLSB

**Figure 6:**
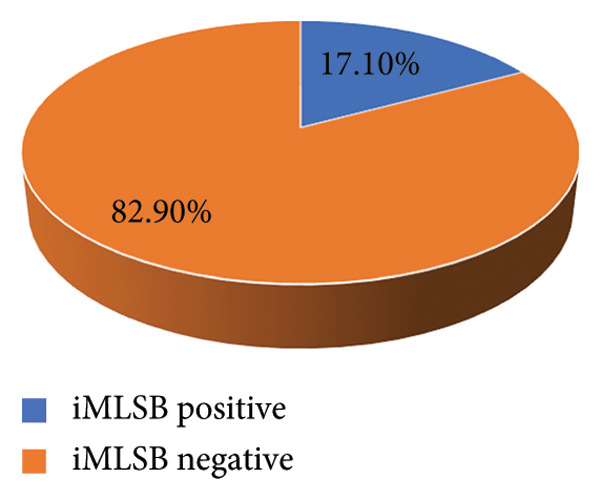
Distribution of iMLSB.

The inducible clindamycin test was found to be positive in 17.1% (37/100) and negative in 82.9% (180/217), which is represented in Figure 6.

### 3.3. Association of Variables With MRSA

Table 2 represents that age (*p* = 0.042), gender (*p* = 0.052), and health faculties (*p* = 0.015) are significantly associated with MRSA.

**Table 2:** Association of MRSA among gender, age, and health faculties.

Variables	Methicillin sensitivity		Total	Chi‐square value	*p* value
	MRSA (*n*/%)	MSSA (*n*/%)	*N*		
*Gender*					
Female	51 (33.1)	103 (66.9)	154	3.204	0.052
Male	29 (46.0)	34 (54.0)	63		

*Age group*					
< 20	23 (50.0)	23 (50.0)	46	6.342	0.042
20–30	52 (32.1)	110 (67.9)	162		
> 30	5 (55.6)	4 (44.4)	9		

*Formation and degree*					
BNS	13 (27.1)	35 (72.9)	48	17.486	0.015
B. Pharm	8 (32.0)	17 (68.0)	25		
BPH	14 (33.3)	28 (66.7)	42		
BPT	7 (14.0%)	7 (14.0%)	14		
BSc. MLT	11 (25.6%)	32 (74.4%)	43		
M. Pharm	3 (42.9%)	4 (57.1%)	7		
MPH	14 (66.7%)	7 (33.3%)	21		
MSc. MM and MB	10 (58.8%)	7 (41.2%)	17		

Abbreviations: MRSA = methicillin‐resistant *Staphylococcus aureus*; MSSA = methicillin‐sensitive *Staphylococcus aureus*.

^∗^Chi‐square test; *p* value ≤ 0.05 considered statistically significant.

## 4. Discussion

An overall *S. aureus* carriage rate of 65.3% was found by Saud et al. in a study involving 352 participants, including medical students and healthcare workers in Kathmandu. Of the isolates, 47.4% were MRSA, and 73.9% were MDR [24]. On the other hand, a 2024 study at Star Hospital in Lalitpur revealed a significantly lower transmission rate, with MRSA isolated in 5.7% of healthcare personnel and *S. aureus* found in 13.3% [22]. *S. aureus* nasal carriage rates were reported to be 88.2%, comparable to 90% in a study conducted among healthcare workers by Khatri et al. [26]. However, the isolation rate of *S. aureus* was lower than our study, with Giri et al. reporting a similar rate of 14.7% [25] and Khatri et al. reporting an isolation rate of 18.3% [26] among healthcare professionals. The research’s overall nasal carriage rate of MRSA was 36.9%, which is comparable to the 35.3% identified in a study conducted by Giri et al. among medical staff in a tertiary care hospital in Kathmandu, Nepal [25]. In contrast, the nasal carriage rate of MRSA in the study conducted by Thapa et al. was greater (39.5%) than the MRSA rate in this study [27]. The nasal carriage rates of MRSA were, however, lower in the studies conducted by Subedi et al. in the tertiary care center and Khanal et al. (2015) in Western Nepal among healthcare workers, at 15.4% [28] and 3.4% [22], respectively, than in this study. Various studies have found different incidence rates, which may be explained by variations in infection control, scheduling, ambient conditions, seasonal variations in hospital visits, job design, and hygiene behaviors [28].

In this study, MRSA in males was found to be 46%, which is greater than that of females (33.1%). Neupane et al.’s investigation also revealed that males had a greater nasal carriage rate of MRSA (67%) than females (33%) [28]. Similarly, in Argentina, Boncompain et al. found that males had a greater rate of MRSA colonization (7.2%) compared to females (5.8%) [29]. However, according to Shakya B et al. (2010), the female MRSA carriage rate was greater (62.5%) than the male MRSA carriage rate (37.5%) [30], which runs counter to what we found.

Students over the age of 30 had the highest MRSA carriage rates (55.6%), followed by those under 20 (50.0%) and those between 20 and 30 (32.1%). In addition, there were notable variations between colonization and age group (*p* < 0.05). The prolonged time of hospital exposure compared to other age group students may be the cause of the greater prevalence of MRSA in the over‐30 age group.

MRSA rates were significantly higher among postgraduate students in MPH and MSc. MM and MB programs in this study, which may be due to different hygiene standards or more clinical exposure. The BNS program’s medical and health science students who must start clinical postings early, even as early as the first year, and have direct contact with patients admitted to Pokhara Academy of Health Sciences hospitals are more likely to come into direct contact with MRSA carriers. Similarly, the prevalence of MRSA infection among BNS was found to be 13 (27.1%). While 92.2% and 87.1% sensitivity to amikacin and tetracycline were seen in our study, a greater proportion of resistance to antibiotics such as erythromycin (74.2%) was followed by amoxicillin (51.6%), clindamycin (42.9%), and ciprofloxacin (30.4%). The susceptibility of all *Staphylococcus aureus* to vancomycin was discovered. MRSA accounted for 36.9% and MDR for 51.6% of the 217 *S. aureus*. According to a study by Thapa et al., all 38 *S. aureus* isolates were resistant to ampicillin and penicillin‐G, susceptible to vancomycin, and of the 38, 50% were MRSA and 67.5% were MDR [27].The D‐zone test was used to check for inducible resistance to clindamycin in *S. aureus* that had demonstrated resistance to erythromycin. Of the 217 *S. aureus* strains in our investigations, 37 (17.1%) exhibited an inducible clindamycin resistance trait. In contrast to other studies, the study conducted by Adhikari et al. found that the prevalence of iMLSB was 11.4%, which is lower than our study [31], and the study conducted by Pradhan et al. found that 39.7% isolates exhibited iMLSB phenotype, which is higher than our study [32].

## 5. Conclusion

According to this study, paramedical students had higher nasal carriage rates of MRSA and *S. aureus.* In addition, MRSA strains showed greater iMLSB resistance than MSSA bacteria. The high rate of MRSA nasal carriage among paramedical students suggests that basic infection control measures should be used in practice to reduce both the carriage and the rate of transmission.

**Nomenclature**
AST: Antibiotic susceptibility testing
BNS: Bachelor in Nursing Science
B. Pharm: Bachelor in Pharmacy
BPH: Bachelor in Public Health
BPT: Bachelor in Physiotherapy
BSc. MLT: Bachelor of Science in Medical Laboratory Technology
DNase: Deoxyribonuclease
HCWs: Healthcare workers
iMLSB: Inducible macrolides, lincosamide, and streptogramin B
MRSA: Methicillin‐resistant *Staphylococcus aureus*
MSSA: Methicillin‐sensitive *Staphylococcus aureus*
MDR: Multidrug resistant
MSA: Mannitol salt agar
M. Pharm: Master in Pharmacy
MPH: Master in Public Health
MM: Medical Microbiology
MB: Medical Biochemistry
PU: Pokhara University
PBP: Penicillin‐binding protein
SHAS: School of Health and Allied Science
SCC: Staphylococcal chromosomal cassette
VRSA: Vancomycin‐resistant *Staphylococcus aureus*
VSSA: Vancomycin‐sensitive *Staphylococcus aureus*

## Ethics Statement

The research has complied with all the relevant national regulations and institutional policies and has been approved by the Institutional Research Committee (IRC) of Pokhara University Research Center, Nepal (letter of approval Registration No: PU‐IRC 61).

## Consent

Written informed consent was taken from all the participants involved in the study.

## Conflicts of Interest

The authors declare no conflicts of interest.

## Funding

No funding was received for this study.

## Data Availability

The datasets of the current study will be available from the corresponding author upon reasonable request.
